# Indigenous Youth Peer-Led Health Promotion in Canada, New Zealand, Australia, and the United States: A Systematic Review of the Approaches, Study Designs, and Effectiveness

**DOI:** 10.3389/fpubh.2018.00031

**Published:** 2018-02-13

**Authors:** Daniel Vujcich, Jessica Thomas, Katy Crawford, James Ward

**Affiliations:** ^1^Aboriginal Health Council of Western Australia, Perth, WA, Australia; ^2^University of Western Australia, Perth, WA, Australia; ^3^University of Notre Dame, Fremantle, WA, Australia; ^4^South Australian Health and Medical Research Institute, Adelaide, SA, Australia; ^5^Flinders University, Adelaide, SA, Australia; ^6^Kimberley Aboriginal Medical Service, Broome, WA, Australia

**Keywords:** peer education, health promotion, Aboriginal health, first nations health research, Indigenous health, systematic review, youth, young people

## Abstract

**Background:**

Youth peer-led interventions have become a popular way of sharing health information with young people and appear well suited to Indigenous community contexts. However, no systematic reviews focusing on Indigenous youth have been published. We conducted a systematic review to understand the range and characteristics of Indigenous youth-led health promotion projects implemented and their effectiveness.

**Methods:**

A systematic search of Medline, Embase, and ProQuest Social Sciences databases was conducted, supplemented by gray literature searches. Included studies focused on interventions where young Indigenous people delivered health information to age-matched peers.

**Results:**

Twenty-four studies were identified for inclusion, based on 20 interventions (9 Australian, 4 Canadian, and 7 from the United States of America). Only one intervention was evaluated using a randomized controlled study design. The majority of evaluations took the form of pre–post studies. Methodological limitations were identified in a majority of studies. Study outcomes included improved knowledge, attitude, and behaviors.

**Conclusion:**

Currently, there is limited high quality evidence for the effectiveness of peer-led health interventions with Indigenous young people, and the literature is dominated by Australian-based sexual health interventions. More systematic research investigating the effectiveness of peer-led inventions is required, specifically with Indigenous populations. To improve health outcomes for Indigenous youth, greater knowledge of the mechanisms and context under which peer-delivered health promotion is effective in comparison to other methods of health promotion is needed.

## Introduction

Improving the health status of Indigenous young people remains a longstanding aspiration in the colonized western countries of Australia, New Zealand, Canada, and the United States of America. Indigenous populations tend to have a younger age profile, and Indigenous adolescents bear a high burden of health problems associated with substance misuse, violence, trauma, sexually transmissible infections and unplanned, high-risk pregnancies; this is related to both historical and contemporary trauma including intergenerational trauma (1).

Government strategies to improve the health outcomes of Indigenous youth often promote the use of peer-led interventions (2–5). Peer-led health promotion is defined as “the teaching or sharing of health information, values and behaviors by members of similar age or status groups” (6). The perceived advantages of the approach derive from the fact that peers have a high level of interaction with one another, and the ability to impart health information in relatable ways (7). Theories of behavioral change (i.e., social learning theory, theory of reasoned action, and diffusion of innovation theory) posit that individuals can be motivated to change their beliefs and practices by observing and interacting with others in their community (8).

Within youth communities, sites of interaction include schools, sporting and recreational events, and designated youth spaces, such as drop-in centers and residential colleges. Interactions can occur one-on-one or in group settings and can take the form of informal discussions between peers or can be more structured. Peer-led interventions are considered particularly useful for educating youth about sensitive topics that may cause fear or embarrassment if discussed with adults, including substance use and sexual health (2).

A number of systematic reviews have examined the efficacy of youth peer-led health promotion programs (9–11). A review by Harden and colleagues found 12 methodologically sound outcome evaluations of peer-led youth health promotion programs; of these, 7 studies found evidence of improved behavioral outcomes (e.g., reduced smoking prevalence, increased frequency of cancer self-examination, reduced incidence of unprotected sex) (9). A further three studies of peer-led interventions found evidence of improvements in relation to “proxy” outcomes, including self-efficacy in using condoms, future intention to use condoms, and attitudes toward sexual health testing (9). A more recent systematic review focusing exclusively on peer-led sexual health interventions supported these findings, with the majority of studies demonstrating improvements in knowledge and attitudes (10). Similarly, a 2016 review of peer-led youth interventions relating to alcohol and other drug use found evidence of lower substance use, improved self-efficacy to engage in safer behaviors, and improved knowledge about target behaviors (11).

However, these studies focus predominately on non-Indigenous populations. Interventions that are effective in one setting are not necessarily directly applicable or transferable to other settings. Effectiveness may be influenced by a range of local factors including social acceptability, culture, the availability of human, financial and material resources, and the educational and socio-economic level of the target population (12). Many of these factors are relevant to Indigenous populations given their unique cultural identities, and experiences of colonization and contemporary social marginalization. For instance, a study of brief intervention tobacco cessation training for clinical staff in an Indigenous health service found no evidence of effectiveness, despite strong evidence from other populations (13). Potential explanations for the difference in outcomes include the fact that health workers were conscious that smoking served an important social function in the communities, and tobacco control was perceived to be less urgent than other local health and social issues (13).

This study seeks to address the gap in the literature by systematically reviewing studies of Indigenous peer-led health promotion programs in Australia, Canada, New Zealand, and the United States of America.

The findings of the study will be used to inform the development of a peer-led program to reduce the rates of sexually transmissible infections and blood-borne viruses among Indigenous youth living in remote Australia (14). To that end, existing studies will be analyzed to ascertain: (1) what approaches to peer-led health promotion have been used in Indigenous contexts; and (2) what is the effectiveness of the different approaches.

## Materials and Methods

Existing systematic reviews on the subject of peer-led health interventions were used to identify potentially relevant search terms (11, 15, 16). A combination of text words and database-specific indexing terms/subject headings were used to increase search sensitivity, and no publication date filters were imposed. The searches were conducted in December 2016 and repeated in June 2017. Full search terms are set out in Supplementary Material.

A combination of health/medical and social science databases (Medline, EMBASE, and ProQuest Social Sciences Database) were searched to reflect the multidisciplinary nature of the study of peer-led youth health interventions. To capture gray literature and publications not contained in electronic databases, supplementary searches were conducted. Google [terms: peer-education AND (young or youth) AND (Indigenous OR Aboriginal) AND health] and Australian Indigenous Health*InfoNet* Health*Bulletin* (terms: peer OR youth OR young) were searched (no comparable Indigenous databases in New Zealand or North America were identified). Only the first 10 pages of results were manually scanned for relevance. Reference lists of included studies were also scanned for relevant literature.

Results were exported to EndNote and titles and abstracts were screened against the inclusion and exclusion criteria. To be included in the systematic review, studies needed to relate to a health promotion intervention that was both aimed at, and delivered by, young people aged 13–29 years who were Indigenous to New Zealand, Australia, Canada, or the United States of America. This systematic review was designed to include both qualitative and quantitative study designs to ensure that both stakeholders’ perceptions/experiences and numerical indicators of effectiveness were captured. Exclusion criteria are set out in Box 1.

Box 1 **Exclusion criteria.**

Exclude publications that:

- are duplicates
- merely describe an intervention without results (e.g., study protocols, program descriptions)
- do not contain a detailed description of study design and/or findings (e.g., conference posters)
- are published in a language other than English.

The titles and abstracts of all studies were screened by one reviewer (Daniel Vujcich for studies published before December 2016; Jessica Thomas for studies between December 2016 and June 2017). All studies which were not excluded upon preliminary review were then independently screened by two reviewers (Daniel Vujcich and Jessica Thomas) with reference to the full text. Inter-coder consistency was high; only two studies resulted in disagreement about application of inclusion/exclusion criteria and the disagreement was resolved following a discussion between the reviewers.

Included studies were coded for details of study population, study design, nature of intervention, and intervention effectiveness. The quality of the included studies was assessed using Critical Appraisal Skills Program Checklists, and major limitations are set out in the Results section. Meta synthesis was not conducted due to the diversity in the design of the included studies.

## Results

**Figure 1:**
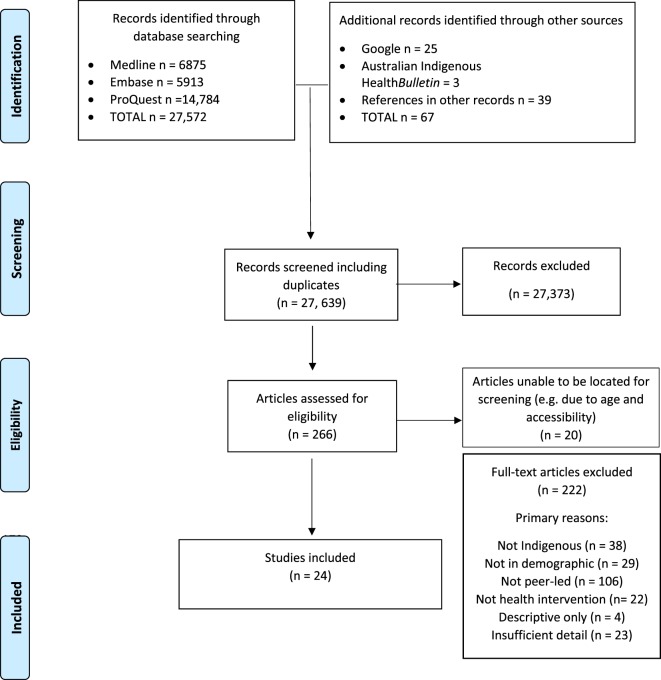
Search result PRISMA diagram (17).

Figure 1 summarizes the results of each stage of the search strategy described above. The 24 included studies related to 20 interventions with Indigenous peer-led components; some studies examined the same programs using different methods or with a focus on different outcomes.

The main characteristics of the interventions are summarized in Table 1. Of the 20 interventions, 9 were based in Australia, 4 were based in Canada, and 7 were based in the United States of America; none of the included studies related to a program aimed at New Zealand’s Maori population. Seven interventions focused on sexual health in combination with another topic area (i.e., alcohol and other drugs/chronic disease/life skills), two focused on sexual health only, three focused on alcohol and other drugs, three studies focused on mental health, two studies focused on asthma/smoking prevention, two studies focused on diabetes, and one focused on cancer prevention generally.

**Table 1:** Characteristics of included studies.

Country	Project	Theme	Setting	Intervention characteristics
Australia	Deadly Liver Mob (17)	Sexual healthAOD	Urban Indigenous and non-Indigenous Clinic-based	Needle and syringe program clients incentivized to recruit and educate Aboriginal peers to attend service

	Young Person Check (18)	Sexual health Chronic disease	Rural Indigenous specific Clinic-based	Community wide “Young Person’s Check” with peer educator-provided health messages and recruiter incentives

	Torres Indigenous Hip Hop Project—Far North Queensland and Torres Strait (19, 20)	Sexual health	Rural Indigenous specific School-based Community-based	Dance and song-writing workshops incorporating sexual health and targeted health messages

	Indigenous Hip Hop Projects—Western Australia (21)	Mental health	Rural Indigenous and non-Indigenous School-based Community-based	Fusion of hip-hop, dance, and cultural workshops with health messages

	Young Nungas Yarning Together (22)	AOD	Urban Indigenous specific Community-based	Peer educator skills development and resource development. Pathway for future accredited training

	Alive and Kicking Goals (23)	Mental health	Rural Indigenous and non-Indigenous Community-based	Football-based peer education training and youth committee

	South Eastern Sydney Division of General Practice Demonstration Project (24)	Sexual healthAOD	Urban Indigenous specific Community-based	Peer educators trained and then delivered messages opportunistically and at request *via* outreach activities

	Indigenous Peer Education Project (25)	Sexual healthLife skills	Urban Indigenous specific Community-based	Three separate peer education projects run by Indigenous staff and wider skill development such as public speaking, first aid, and computing skills

	Peer-Led Asthma and Smoking Prevention Project (26)	AsthmaSmoking	Urban Indigenous and non-Indigenous School-based	Tiered workshops with teachers and students, with subsequent training delivered by previous workshop participants

Canada	Taking action against HIV (27–29)	Sexual healthAOD	Rural and urban Indigenous specific Community-based	Sexual health workshops facilitated by a local youth coordinator and supported by elders

	Beating Diabetes Together (30)	Diabetes	Rural/urban status not specified Indigenous specific Community-based	Weight loss curriculum delivered by university students in an after school setting

	HIV/AIDS education program, Ontario (31)	Sexual healthAOD	Rural/urban status not specified Indigenous specific Community-based	Community facilitators recruited and trained. Facilitators then recruited volunteer workshops and delivered training

	Fourth R (32)	Mental health	Rural/urban status not specified Indigenous and non-Indigenous School-based	Young adults deliver an 18-week course to upper-elementary school students, based on the Indigenous Medicine Wheel Life Cycles

USA	Native STAND (Students Together against Negative Decisions) (33, 34)	Sexual health	Rural/urban status not specified Indigenous specific School-based	Peer- and self-nominated participants attended 29 sessions

	The Native Comic Book Project (35)	Cancer prevention	Rural/urban status not specified Indigenous specific Community-based	Youth leaders trained to plan, write and design original comic books to enhance healthy decision-making for cancer reduction

	Narragansett Substance Abuse Prevention (36)	AOD	Urban Indigenous specific Community-based	Youth participants received training as peer assistant leaders in an ongoing community drug abuse prevention project

	Youth Services Program (37)	Sexual healthAOD	Urban Indigenous specific Community-based	Youth services hosted traditional and contemporary Native cultural activities (e.g., dance and art) with alcohol and drug messages as a part of a wider event

	STOP Diabetes! (38)	Diabetes	Rural Indigenous specific Community-based	Workshop and manual developed for youth participants based on nutrition and physical activity in a cultural context

	Peer-Managed Self-Control Program for Prevention of Alcohol Abuse (39)	AOD	Rural/urban status not specified Indigenous specific School-based	Youth met with peer counselors who instructed students in self-monitoring and assisted them to set up self-contracts with respect to alcohol consumption

	Crossroads (40)	Smoking prevention	Rural/urban status not specified Indigenous and non-Indigenous School-based	Youth participated in focus groups to workshop ideas for a tobacco prevention play. A young person wrote a script based on the focus group discussion. The play was performed at elementary and middle-schools

*AOD, alcohol and/or other drugs*.

Six programs were conducted in rural and/or remote settings (one of these also had an urban component) and seven projects were aimed at urban youth only. The setting was not reported for the remaining seven interventions.

Eleven of the interventions were community-based only (and two were a combination of community- and school-based). The majority of community-based interventions used cultural and artistic activities as a means of engaging youth (19–22, 27–29, 37). For instance, in some Australian projects, Indigenous youth taught dance, song-writing, and video skills to peers and encouraged them to develop products and/or performances that could be used for wider health promotion (19–22). Similarly, in the United States, the Native Comic Book Project trained young people to deliver 16 lessons to peers with the goal of creating comic books to enhance healthy decision-making around cancer prevention (35).

Five of the interventions were school-based only. These varied between structured curricula/classes (26, 32–34), mentoring arrangements in which older youth offered health guidance and support to younger peers (39) and a creative project whereby students were involved in developing and performing a play incorporating health messages (40).

In the two clinic-based peer-led interventions, youth encouraged their peers to engage with health services. For the Deadly Liver Mob project (17), Indigenous clients attending a needle and syringe program were given a monetary incentive for educating other people about hepatitis C transmission and getting them to visit the service. Similarly, the Young Person Check project (18) offered monetary incentives to youth who recruited peers to obtain STI tests at the clinic.

The interventions differed in terms of the degree of formal education that was offered to peer educators. In some interventions, a highly structured train-the-trainer format was utilized. The Native STAND project (34–35) required peer leaders to complete a course comprising 29 weekly sessions, and the Indigenous Peer Education Project included wider skill development such as public speaking, first aid and computing skills (25). By contrast, other interventions imparted information through one-off sessions and encouraged participants to share what they had learned with others. For instance, peer leaders in the Deadly Liver Mob project received information about blood-borne viruses from Aboriginal health workers during a clinic visit and were encouraged to pass the information on to others (17).

Many of the interventions incorporated some element of Indigenous cultural education or practice. The Four R Program (32) was based on the Indigenous Medicine Wheel Life Cycles, the Indigenous Hip Hop Projects (21) fused traditional culture with modern art forms, and the Taking Action against HIV intervention (29) educated youth about the impact of colonization on Indigenous health outcomes.

The majority of studies found some evidence of changes in behavior, knowledge, or attitude associated with peer-led interventions, as set out in Table 2. Evidence of changes in behavior included increased STI/BBV testing (17, 18), increased use of health services (25), and decreased alcohol and/or other substance use (36, 39). Effects on knowledge included increased awareness of sexual health issues (19, 25, 27–29, 31, 33, 37), improved healthy lifestyle knowledge (38), better understanding of dangers of drug abuse and/or addiction (37, 40), and better understanding about mental health issues and how to support someone feeling depressed (21). Attitudinal changes included improved self-confidence, self-esteem, and/or self-perception (22, 25, 27–29, 31, 32, 35, 36), increased intention to reduce/abstain from substance use (26, 40), and increased intention to use condoms (33).

**Table 2:** Study design characteristics of included studies.

Study	Design	Sample size	Analysis	Select results	Main quality issues/comments
Deadly Liver Mob (17)	Experimental pre–post study using: Attendance data Testing data	Pre-intervention group *n* = 83 Post-intervention group *n* = 306	Chi-squared or Fischer exact tests for differences in distribution of categorical variables *t* or Mann–Whitney *U* tests for differences in distribution of continuous variables	Intervention associated with: increase in clinic visits increase in attendance for asymptomatic STI/BBV screening increase in proportion tested for at least five STIs/BBVs	Number of Indigenous people attending clinic pre-intervention may have been underestimated (staff had increased awareness of need for accurate reporting during intervention) As this is a multicomponent intervention, it is difficult to discern whether the change was attributable to peer-led intervention, or other factors such as financial incentive

Young Person Check (18)	Cross-sectional study using testing data (period prevalence)	Eight discrete communities One community cluster containing five villages Estimated total population of 2,068 Indigenous 15–24 years	Descriptive statistics	3,083 episodes of care Coverage of the 15–24 population in each location ranged from 50 to 87%, with 13 of 19 events achieving target group coverage of at least 70%, and a further five achieving 65–69% On one occasion, participation was below target at 50% Of the five communities that held more than one YPC, one demonstrated a significant upward trend in testing over 5 years	Not possible to isolate impact of peer intervention from financial incentive

Indigenous Hip Hop Project—Torres Strait and Far North Queensland (19, 20)	Case study using: Attendance numbers Interviews Debrief session notes Evaluation report	Unspecified	Content analysis of qualitative data Descriptive statistics for quantitative data	Average 80% of school students participated in workshops 16 songs composed and recorded at workshops “High” attendance numbers for gala events Increased awareness of sexual health disadvantage Local health promotion supported	Recruitment strategy not specified Data collection methods not explicit (e.g., how interviews conducted) Insufficient data presented to support findings (e.g., specific figures, quotations) No in-depth description of data analysis process

Indigenous Hip Hop Projects—Western Australia (21)	Experimental pre–post study using: Interviews Focus groups Field notes Questionnaires	Stage 1 (immediately post-intervention), *n* = 76 Stage 2 (4 weeks post-intervention), *n* = 47 Stage 3 (6 months post-intervention), *n* = 41 youth + unspecified number of school personnel and service providers	Thematic content analysis Descriptive statistics	23% of participants responded that they did not know what to do if someone was feeling down/depressed pre-intervention (the majority of respondents reported knowing what to do after week 1 of the intervention) While there did not appear to be unprompted recall of discussion about depression/anxiety among participants 6 months following IHHP visit they reported “feeling good about themselves” as a result of some of the IHHP activities Young people appear to have some understanding of what depression is, although this appeared to be strongest in the week of the IHHP visit	High loss to follow-up between stage 1 and stage 2 Stage 2 included some participants who did not provide data in stage 1 Limited presentation of data comparing participants’ stated knowledge prior to intervention with knowledge post-intervention (difficult to determine impact of intervention)

Young Nungas Yarning Together (22)	Focus group Interviews	Focus group, *n* = 4 youth Interviews, *n* = 2 staff	No information provided	15 youth completed the course An educational DVD resource was produced by the youth Program increased the confidence of participants	No clear statement of research aims Poor recruitment due to length of time between program and data collection Insufficient data presented to support the findings

Alive and Kicking Goals (23)	Unspecified	Unspecified	Unspecified	16 participants trained as peer educators Peer educators helped to begin community conversations about suicide	Insufficient detail about study design and data Author was closely involved in implementation of the project

South Eastern Sydney Division of General Practice Demonstration Project (24)	Unspecified	Unspecified	Unspecified	All second-year peer educators gained more permanent employment or traineeships	Insufficient detail about study design and data

Indigenous Peer Education Project (25)	Rapid ethnography using: Focus group Interviews Document analysis	Interviews, *n* = 13 youth and staff Focus group, *n* = 4 youth	Unspecified	22/28 peer educators completed program Peer educators reported improved confidence, increased sexual health knowledge, increased short-term use of health services, specialized skills and re-entry to school or work Peer educators competently delivered education sessions Peer educators continued to use skills in peer education in opportunistic manner post-intervention A formal ongoing network of peer educators did not materialize	Pre- and post-knowledge surveys were administered to participants but “these data were not available for analysis”

Asthma and Smoking Prevention Program (26)	Experimental pre–post study using: Questionnaires Exhaled carbon monoxide (eCO) testing Peer leaders also completed feedback questionnaire	Questionnaires, *n* = 173 baseline and *n* = 156 at follow-up eCO testing, *n* = 91 baseline and *n* = 77 at follow-up Number of Indigenous respondents unspecified	Descriptive statistics Thematic content analysis for qualitative questionnaire data	Of the three reported smokers at baseline, only one had elevated CO levels at follow-up No reduction in self-reported asthma at follow-up Smoking pledge signed by 49% of participants Peer leader feedback was overwhelmingly positive	Sample too small to examine for differences in asthma control and uptake of tobacco smoking Questionnaires were reported to be unsuitable for this population due to language used

Taking action against HIV (27–29)	Interviews	*n* = 70	Thematic content analysis	Prior to attending workshop many youth were unaware of HIV and its prevalence in Aboriginal communities Workshop cleared up myths and misconceptions around HIV Youth regarded the arts-based process as fun, participatory, self-esteem enhancing and healing The process enhanced recall and facilitated dialog on sensitive subjects	Insufficient detail about study design (interview questions, etc.) Participants were self-selected which may introduce bias

Beating Diabetes Together (30)	Experimental pre–post study using: Anthropometric measurements Glycated hemoglobin Interviews also conducted	Quantitative arm, *n* = 12 Qualitative arm, *n* = 5 youth, *n* = 2 mothers	Paired *t*-tests to describe changes in outcome measures post-intervention Thematic content analysis of qualitative data	Glycemic control, blood pressure and anthropometric measures unchanged All participants described enjoying the intervention Intervention was well-attended One participant explained how participation gave her hope about her illness	Small sample Insufficiently powered Confounding factors not considered in design

Ontario HIV/AIDS education program (31)	Experimental pre–post study using questionnaires Evaluation forms	*n* = 24	Unspecified	Increase in level of HIV/AIDS knowledge Increase in self-confidence Increase in self-esteem Change in group attitude toward HIV/AIDS	Insufficient detail about study design and data

Fourth R (32)	Prospective cohort study using: Surveys Interviews	Surveys, *n* = 105 (exposed group, *n* = 18, unexposed group, *n* = 85) Interviews, *n* = 28	Descriptive statistics Thematic content analysis of qualitative data *t* tests Chi-squared tests	Participants who received 2 years of mentoring reported greater cultural identity and better mental health than those who received less/no mentoring Participants expressed that the intervention has a positive impact on personal growth, self-confidence	Small sample Underpowered to account for school-level differences

Native STAND (Students Together against Negative Decisions) (33, 34)	Experimental pre–post study using survey Interviews Focus groups	Pre-intervention, *n* = 70 Post-intervention, *n* = 34 Details of qualitative sample not reported here	Cohen’s *d*	Increase in: students who reported that they had talked to a peer about sexual health in past 3 months; STI/HIV prevention knowledge; reproductive health knowledge; knowledge of health relationships; intention to use condoms No significant changes in self-esteem, motivation to be a role model or self-efficacy for being a peer educator	High loss to follow-up

	Experimental pre–post study using survey Interviews Focus groups	Survey, *n* = 90 youth Interviews, 9 key informants Focus groups, *n* = 129 youth	Unspecified for quantitative data Grounded theory methods for focus group data	Observed improvements in behaviors associated with STI risk were not statistically significant Students perceived that arts-based methods improved retention, self-esteem, and self-confidence	Insufficient detail about qualitative study design and data Insufficiently powered

Native Comic Book Project (35)	Experimental pre–post study using survey Interviews Focus groups Comic strip evaluation tool	*n* = 6 youth Sample size unspecified for other types of participants (e.g., trainers, parents)	Unspecified	Post-intervention youth were more likely to indicate strong agreement with statement “Young people can make a difference in their community” Youth enthusiastic about implementing project in communities	Small sample size and anonymity meant pre- and post-comparisons were not possible Insufficient detail about study design and data

Narragansett Substance Abuse Prevention (36)	Non-randomized case–control study using: Surveys Interviews Field notes	Control group, *n* = 25 (11 did not complete program) Intervention group, *n* = 9	Unspecified	Individuals in intervention group stated that they stayed in the program largely due to the cultural material Reported reduction of drug use between groups (but not quantified) Correlation between increased cultural affiliation and decreased substance use Individuals in intervention group reported more positive self-perceptions	Insufficient detail about study design and data Confounding factors not considered in design (control group included many non-Indigenous youth) High loss to follow-up in control group Subjective (self-reported) measurements of substance use

Youth Services Program (37)	Survey	*n* = 34 (23 Indigenous)	Unspecified	80% reported more knowledge of HIV as a result of intervention 86% reported more knowledge about dangers of unsafe sex as a result of intervention 83% reported more knowledge about dangers of drug abuse and addiction as result of intervention	Sampling strategy not specified Insufficient detail about study design Subjective (self-reported) measurements of intervention impact

STOP Diabetes! (38)	Experimental pre–post study using: Questionnaires Evaluation forms Anthropometric measurements also taken at time of intervention (but not post-intervention)	Questionnaires, *n* = 9 Evaluation forms, unspecified	Improved score calculated for questionnaires Each of the four dichotomous questions in the evaluation were worth two points if answered “yes” (a sum of ≥4 was interpreted as a positive experience)	89% of complete test sets demonstrated improved knowledge post-intervention 90% of participants reported a positive workshop experience	Small sample Low response rates (38% for complete pre/post questionnaires)

Peer-Managed Self-Control Program for Prevention of Alcohol Abuse (39)	Randomized control trial using: Questionnaires Blood-alcohol testing	Group A (classes, peer counseling and self-contracts), *n* = 12 Group B (peer counseling with self-contracts), *n* = 8 Group C (peer counseling only), *n* = 10	ANCOVA	All groups decreased weekly quantity and frequency of drinking over time at all follow-up points No differential change observed among groups regarding quantity consumed Differential change observed among groups regarding drinking frequency, with greatest improvement in Group C No changes in knowledge or attitudes about alcohol from pre- to post-intervention	Small sample Study did not have a no-treatment group (all groups received some sort of intervention)

Crossroads (40)	Experimental pre–post study using questionnaires	*N* = 2,660 (295 Native Hawaiian)	McNemar tests	After watching the play, students were more likely to: understand addiction; correctly define second-hand smoking; report future intentions to avoid smoking	Possibility of bias because sample represents only ~25% of people who viewed play

The quality of the evidence was variable. The only randomized controlled trial was a study in which American Indian teenagers were randomly assigned to one of three group interventions designed to prevent alcohol abuse (39). All groups involved some element of peer counseling, but differed in terms of their additional components (one group had no additional components, one group included self-contracts establishing limits on alcohol use, and the final group included self-contracts and classes). The quantity and frequency of drinking decreased for all groups; however, the results were derived from a small sample (30 youth across the three groups) and the absence of a “non-treatment” group makes it more difficult to discount the possibility that external factors may have driven the observed change. Similarly, the results from a non-randomized trial (the Narragansett substance abuse prevention program) were limited by the fact that the samples were small (*n* = 9 in intervention and *n* = 25 control), and confounding factors were not considered in the design despite significant differences in the demographic characteristics of the groups (36).

The majority of the remaining publications were based on experimental pre- and post-study designs. The validity of the results were affected by methodological limitations including small samples (26, 30, 32, 35, 38, 39); high loss to follow-up (21, 33, 36); limited presentation of data (19–25, 29, 31, 34–37); difficulties disaggregating the effects of peer-led interventions from simultaneous interventions (17, 18); and failure to account for confounding factors (30).

## Discussion

This review investigated the use and effectiveness of peer-led health promotion by Indigenous youth. Twenty examples of youth peer-led health interventions in Indigenous contexts were found. The interventions included in this systematic review were most commonly on the topic of sexual health, alcohol, and other drugs and mental health/suicide prevention. Most interventions were based in Australia. Only a minority of studies found evidence of changes of behavior, although this is common in evaluations of public health interventions given the need for long follow-up periods (42). Evidence of changes in knowledge and attitudes was more common, consistent with systematic review findings on the effectiveness of peer-based interventions in other settings (10, 43). In addition to population health improvements, benefits were also conferred to peer leaders in the form of improved self-perception and, in one case, post-intervention employment opportunities.

Methodological limitations impacted on the quality of evidence-base relating to peer-based interventions for Indigenous youth. The relative dearth of “high level” evidence on this subject is not surprising. There are a number of difficulties associated with evaluating peer-led interventions involving Indigenous youth. First, any research involving youth raises distinct ethical issues; these include perceived power disparities, capacity to provide informed consent and legal obligations on the researcher to disclose otherwise confidential data (e.g., reports of physical or sexual abuse) (44, 45). Parents, schools, and other authorities often act as gatekeepers, thus limiting researchers’ access to young people (44–46). Consequently, researchers may avoid studying young people in favor of other classes of participants.

Second, researchers may have difficulty recruiting sufficiently large samples of Indigenous people in the relevant demographic, as shown in Table 2. Given their experiences of colonial exploitation, some Indigenous communities are wary of research and individuals may be reluctant to participate in studies (47–49). Moreover, Indigenous people comprise only a small proportion of the total population in Australia (3%), New Zealand (15%), Canada (4%), and the United States of America (1%) (50–53). It follows that:

many data sources are unsuitable for Indigenous program evaluation because they do not have sufficient numbers of Indigenous respondents for analysis. Even when quantitative analysis is possible, small sample sizes can drastically limit statistical power. This means that, given realistic sample sizes, only very large program impacts are likely to be detected at standard statistical levels (54).

Other issues which can affect research in Indigenous contexts include remoteness, transient populations, and delays due to cultural events (55).

Finally, there are a number of barriers to accurately gauging the effects of population or community-level interventions, regardless of the target group. For example, it can be difficult to recruit sufficient numbers of communities with comparable characteristics; replicate the level and intensity of exposure across communities; and ascertain whether any observed changes are attributable to the intervention or other environmental influences (56).

It does not follow that research on the effectiveness of Indigenous peer-led health interventions should be dismissed as being too difficult. A number of high quality randomized controlled trials have been conducted to evaluate the effectiveness of peer-led interventions among Indigenous children (below the 13- to 29-year-old age category that is the focus of this review). These include an evaluation of the Healthy Buddies program in Manitoba in which 60 schools were enrolled in the study; 10 schools were randomly assigned to the Healthy Buddies program, and 10 schools were assigned to receive a standard curriculum (57). First Nations schools were equally represented in the intervention and control arm. Students receiving the peer-led intervention had a significant reduction in waist circumference compared with the control group, and the effects on waist circumference were higher among First Nations compared with other students. Rigorous school-level non-randomized case–control studies of interventions for Indigenous Canadian children have also been conducted and have demonstrated significant effects on physical and behavioral outcomes (58, 59).

In addition to research conducted in academic settings, providers of peer-led health programs could be empowered to build the evidence base. Recommendations include improving service providers’ access to practical evaluation tools; developing their knowledge and skills in evaluation techniques; and providing additional funding to support rigorous data collection (60).

There is also a need for studies which directly compare whether peer-led health interventions are more effective if delivered in school or non-school settings, or whether certain features such as length of training, cultural content, or provision of incentives improve efficacy. At present, funders and planners have little empirical guidance as to what features of peer-led interventions are essential to maximize success. Such information is needed to ensure that resources are utilized in a manner that is most likely to redress the health disparities between Indigenous and non-Indigenous youth. Research to identify factors influencing success is also necessary given the findings that peer-led health promotion can affect young people’s self-esteem and self-confidence.

With respect to the limitations of this review, it is likely that some studies of Indigenous peer-led health interventions were not located because the findings were not publicly available. The search strategy for this systematic review included gray literature; however, it is possible that relevant sources of gray literature from New Zealand and North America were inadvertently missed by the Australian-based researchers. In addition, some potentially relevant studies may have been excluded because there was insufficient detail to determine whether the inclusion criteria were met.

## Conclusion

Currently, there is limited evidence for the effectiveness of peer-led health interventions with Indigenous young people and the knowledge base is dominated by Australian-based sexual health interventions. The studies found positive outcomes from youth peer-led interventions; however, the research available has methodological limitations. More systematic research investigating the effectiveness of peer-led inventions, particularly with Indigenous populations, is required. To improve health outcomes for Indigenous youth, greater knowledge of the mechanisms and context under which peer-delivered health promotion is effective in comparison to other methods of health promotion is needed.

## Author Contributions

DV, JT, and JW contributed to the design of the work. DV and JT acquired data. All authors contributed to analysis and interpretation, contributed to drafting and critical revisions, approved the final version for publication, and agreed to be accountable for all aspects of the work in ensuring that questions related to the accuracy or integrity of any part of the work are appropriately investigated and resolved.

## Conflict of Interest Statement

The authors declare that the research was conducted in the absence of any commercial or financial relationships that could be construed as a potential conflict of interest.
