# Supplementary material for: Indigenous Youth Peer-Led Health Promotion in Canada, New Zealand, Australia, and the United States: A Systematic Review of the Approaches, Study Designs, and Effectiveness
Source: Front Public Health. 2018 Feb 13;6:31. doi: 10.3389/fpubh.2018.00031 (PMC5818867; doi:10.3389/fpubh.2018.00031)
Supplement: Supplementary file 1 [file Table_1.docx]

**Supplementary Materials**

| **Database** | **Search strategy** |
| --- | --- |
| Medline | ((aboriginal.mp. or exp indigenous people/) OR (exp Aborigine/ or exp Torres Strait Islander/ or torres strait.mp.) OR (indigenous.mp. or exp Indigenous Australian/) OR (first nations.mp. or exp First Nation/) OR (maori.mp. or exp "Maori (people)"/) OR (exp American Indian/) OR (metis.mp. or exp Metis/ or exp Eskimo/) OR (inuit.mp. or exp Inuit/)) **AND** ((exp peer counseling/ or peer*.mp. or exp peer group/) OR (adolescent/) OR (youth.mp. or exp juvenile/) OR (exp adolescence/) OR (young.mp. or exp young adult/) OR (exp student/ or student.mp.) OR (pupil.mp.)) **AND** ((leader.mp. or exp leadership/) OR (support.mp. or exp social support/) OR (ministry.mp.) OR (ambassador.mp.) OR (health worker.mp. or exp health care personnel/) OR (outreach.mp.) OR (health promotion.mp. or exp health education/ or exp health promotion/) OR (exp teaching/ or teach*.mp.) OR (exp social network/ or network*.mp.) OR (exp counseling/ or counsel*.mp.) OR (exp education/ or educat*.mp.)) |
| EMBASE | ((aboriginal.mp. or exp indigenous people/) OR (exp Aborigine/ or exp Torres Strait Islander/ or torres strait.mp.) OR (indigenous.mp. or exp Indigenous Australian/) OR (first nations.mp. or exp First Nation/) OR (maori.mp. or exp "Maori (people)"/) OR (exp American Indian/) OR (metis.mp. or exp Metis/ or exp Eskimo/) OR (inuit.mp. or exp Inuit/)) **AND** ((exp peer counseling/ or peer*.mp. or exp peer group/) OR (adolescent/) OR (youth.mp. or exp juvenile/) OR (exp adolescence/) OR (young.mp. or exp young adult/) OR (exp student/ or student.mp.) OR (pupil.mp.)) **AND** ((exp education/ or educat*.mp.) OR (exp counseling/ or counsel*.mp.) OR (exp social network/ or network*.mp.) OR (exp teaching/ or teach*.mp.) OR (health promotion.mp. or exp health education/ or exp health promotion/) OR (outreach.mp.) OR (health worker.mp. or exp health care personnel/) OR (ambassador.mp.) OR (ministry.mp.) OR (support.mp. or exp social support/) OR (leader.mp. or exp leadership/)) |
| ProQuest Social Sciences Database | (Aborigin* OR "Torres Strait" OR Indigenous OR "First Nations" OR MAORI OR "Native American" OR Metis OR Inuit) **AND** (peer* OR adolescent OR youth OR teen* OR young OR student OR pupil) **AND** (educat* OR counsel* OR network* OR teach* OR "health promotion" OR outreach OR ambassador OR ministry OR support OR leader) **AND** (health) **AND** stype.exact("Conference Papers & Proceedings" OR "Scholarly Journals" OR "Reports" OR "Books" OR "Working Papers") AND at.exact("Article" OR "Conference Proceeding" OR "Working Paper/Pre-Print" OR "Review" OR "Book" OR "Government & Official Document" OR "Reference Document" OR "Bibliography" OR "Case Study" OR "Conference Paper" OR "Industry Report" OR "Report" OR "Evidence Based Healthcare" OR "Literature Review") **AND** la.exact("English") |
